# Recreationist willingness to pay for aquatic invasive species management

**DOI:** 10.1371/journal.pone.0246860

**Published:** 2021-04-14

**Authors:** Lucia R. Levers, Amit K. Pradhananga

**Affiliations:** 1 Water Resources Center, University of Minnesota, Minneapolis, Minnesota, United States of America; 2 Center for Changing Landscapes, University of Minnesota, Minneapolis, Minnesota, United States of America; University of Waikato, NEW ZEALAND

## Abstract

We estimated willingness to pay for local aquatic invasive species lake management in the form of a daily lake access fee by conducting summer lake surveys in Minnesota, USA. Similar pairs of lakes with differing infestations of zebra mussels, *Dreissena polymorpha*, and starry stonewort, *Nitellopsis obtuse*, were used as study sites to infer how being at an infested lake vs. being at an uninfested lake and different local species would impact responses. We also examined recreationists’ visit motivation, and aquatic invasive species perceived risk, knowledge, and awareness of problem. We estimated mean willingness to pay about nine to ten dollars per day, which did not differ significantly by lake. Additionally, perceived risk, awareness of problem, and visit motivation were significant in predicting willingness to pay, which could have important ramifications for aquatic invasive species management.

## 1 Introduction

Aquatic invasive species (AIS) are a growing problem in freshwater systems throughout the world, negatively affecting native biota and human populations. The aquatic environment poses some unique challenges to invasive species management, particularly as it relates to detection and control [1–3]. In areas which have high numbers of water bodies and high numbers of water craft (1 out of 6 Minnesotans owns a boat—in more northern areas, it is 1 out of 3 [4]), invasive species are easily moved from one site to the next and have gained substantial foothold. In Minnesota alone, the Department of Natural Resources (DNR) lists 37 different aquatic invasive species [5]. Some of which, like common carp, *Cyprinus carpio*, and curlyleaf pondweed, *Potamogeton crispus*, are considered naturalized and are no longer tracked by DNR. Some have been present for decades, like zebra mussels, *Dreissena polymorpha*. Others are relatively new, like starry stonewort, *Nitellopsis obtuse*. With such a variety of species, it is understandable that effects are as varied as the species themselves. When multiple species are infesting the same water body, impacts become even more difficult to tease out. Combined with climate change, and other anthropogenic influences on environmental quality and ecosystem health, the true impacts of a particular species can be extremely difficult to determine for scientists, let alone laypersons. Ideally, to understand how people value invasive species, we would need to fully understand 1) underlying causal relationships between invasive species and environmental impacts, and 2) how individuals in question value those impacts.

While much attention has been paid to understanding ecological and economic impacts of invasive species, linkages between human systems and ecosystem impacts of invasive species are not well understood. In recent years, there has been an increase in understanding the economic impacts of invasive species using contingent valuation methods (e.g., [6, 7]). Yet, there is little clarity of the underlying motivations for individuals’ support and willingness to pay for invasive management. From a management perspective, understanding people’s motivations for and constraints to actions that prevent or manage the spread of invasive species can help resource managers develop programs that are based on public needs and concerns.

Very few studies have focused on the social-psychological determinants of willingness to pay for invasive species management. In a study of residents, tourists, and conservationists in Spain [8], the authors found that attitudes about invasive species and invasive species management were related to willingness to pay. In a similar study [9], the authors reported that demographic variables such as higher levels of income and smaller household size were positively associated with willingness to pay for invasive species management. Further, reported interest in nature (e.g., membership in environmental organizations), knowledge about invasive species, concern about invasive species impacts on cultural identity, and sense of place (i.e., emotional connection people feel for a geographic area) were positive predictors of willingness to pay. Other researchers have also linked income, interest in nature [6, 10], and age [10] with willingness to pay. While not in the context of invasive species management, a subset of studies have argued that it is critical to examine the underlying social-psychological factors that influence willingness to pay [1, 2]. These studies have generally concluded that the inclusion of social-psychological theories and variables improves the explanatory power of models examining willingness to pay. For example, a study applying different theoretical models to willingness to pay [1] found that personal norm and awareness of responsibility, variables from the norm-activation theory [3], have higher explanatory power than variables derived from theories such as the theory of planned behavior and the theory of public goods. Further, environmental concern has been reported as a significant predictor of willingness to pay for public environmental goods (i.e., forest biodiversity). Another study [2] provides empirical support for the influence of variables from the theory of planned behavior [4] on willingness to pay for increase in biodiversity. Attitudes about biodiversity, subjective norms (i.e., social pressure to take action), and perceived behavioral control (i.e., ease or difficulty of performing a behavior) were significant predictors of willingness to pay [2]. Studies have also linked knowledge, past environmental activism, trust in governing agencies [5], value orientations, awareness [6], perceived effectiveness of policy, social capital [7] and perception of other actors’ actions [5, 7] with willingness to pay for environmental goods in the context of energy consumption, emission reductions, and waste management. This study builds on this body of research by exploring the social-psychological factors that influence willingness to pay for invasive species management. This project employed on-site summer lake surveys to understand how recreationists using public lake access points perceived and valued aquatic invasive species management, and whether different species and magnitudes of infestations (proxied by different lakes) influenced their perceptions of AIS and their willingness to pay for aquatic invasive species management.

## 2 Conceptual framework

This study’s conceptual framework integrated multiple lines of research that link environmental awareness, risk perception, motivations, and environmental behaviors. While environmental behaviors have been studied from a wide range of theoretical perspectives [8], research in the human dimensions of natural resources suggests that a cognitive structure of values, attitudes, and beliefs affect general pro-environmental behaviors, as well as behaviors targeted at invasive species [9, 10]. According to the cognitive hierarchy theory, human cognitions are organized hierarchically from values, which are centrally held and stable, to elements such as attitudes, beliefs, and ultimately behaviors, which are numerous and more easily changed [11, 12]. This framework has been applied in various natural resource contexts [11–14]. We build on the cognitive hierarchy theory framework by integrating it with risk perception theory.

Researchers have extensively studied risk perceptions and its influence on environmental behavior [15–17]. Risk perception is defined as the process of “discerning and interpreting signals from diverse sources regarding uncertain events and forming a subjective judgement of the probability and severity of current or future harm associated with these events” ([15], p. 1). The concept of risk perception has been applied extensively to behaviors related to climate change. For example, a nationwide study of US residents found a significant influence of risk perception on voluntary climate change action (e.g., choice of transportation) and voting intentions (e.g., support for climate change-related government programs) [18]. Similarly, recent studies have reported risk perceptions as determinants of energy conservation [19], general pro-environmental behaviors (e.g., recycling, buying organic food) [15], willingness to participate in organic farming programs [20], and travel behavior [21]. While literature linking risk perception to invasive species prevention and control behaviors is scant, Estévez et al. [9] provide an integrated risk perception and cognitive hierarchy theory framework to study the human and social dimensions of invasive species management.

We also included knowledge about AIS and awareness of AIS problem as determinants of willingness to pay in our framework. While some studies have found weak to no relationship between knowledge and environmental behavior [22], a few studies provide support for the relationship between knowledge and AIS control behaviors, e.g. [23], as well as willingness to pay for environmental protection in the context of invasive species [24], greenhouse gas emissions [5], and stormwater management [25, 26]. Thus, some knowledge and awareness about environmental issues may be necessary for environmental actions [27].

Past work, particularly in the area of leisure and recreation management, has investigated people’s motivations for engaging in leisure and recreation [28, 29]. The question of ‘why’ people engage in recreation or the desired outcomes of engaging in recreation (i.e., visit motivation) has been linked with recreationist behaviors, preferences, and satisfaction [29–31]. Since the survey sample in our study consisted of recreationists intercepted at lakes, it is plausible to hypothesize that their motivations for visiting the lake would have an influence on the actions aimed at protecting the lake from AIS. Thus, we included visit motivation in our conceptual framework as a cognitive element that influences recreationists’ willingness to pay for AIS management.

## 3 Study sites

Priority species for management in Minnesota include zebra mussels, *Dreissena polymorpha*, and starry stonewort, *Nitellopsis obtuse*. Zebra mussels attach themselves to a myriad of surfaces in lakes, smothering native species, increasing water clarity, and causing property damages [18]. Additionally, walleye, *Sander vitreus*, a recreationally and ecologically important fish species in North America, grow more slowly in their first year when zebra mussels are present [19]. Starry stonewort is a macro-algae that forms thick mats just below the lake surface, reducing navigability and impacting native species [20]. We chose two pairs of lakes, with each pair representing a spectrum of infestation for each priority species, yet being as similar as possible in other measures such as limnological factors, typical recreational use, size, proximity, public access similarity, and nearby human population types. Sampling locations at each lake were public lake access points managed by the Minnesota Department of Natural Resources. They all included a boat ramp, parking facilities, and restrooms. A special use permit covering all locations and dates was obtained from the Minnesota Department of Natural Resources Division of Parks and Trails prior to data collection.

**Fig 1:**
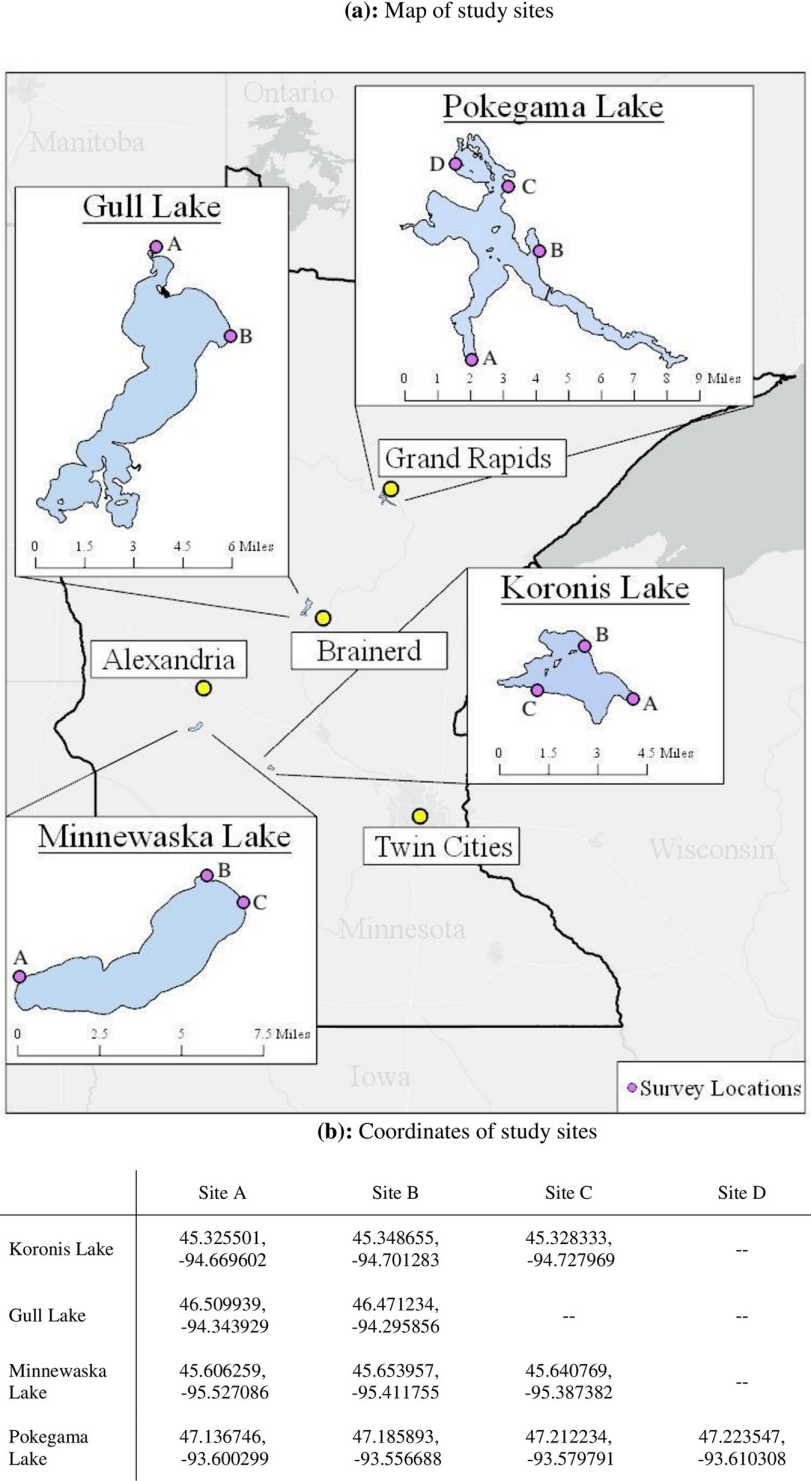
Location of study sites in the state of Minnesota.

Because we knew that the public may not be aware of the local AIS, we chose lakes that had very obvious water quality impacts of infestation. We also needed to ensure the lakes had similar public access quality, visitation rates were high enough for adequate sampling, and preferably that the lakes were close together. Though, because of the nature of AIS spread, lakes nearby to one another are often infested with the same species. With consultation and data of the Minnesota Department of Natural Resources, expert opinion of Minnesota Aquatic Invasive Species Center affiliates, and ground-truthing, we chose the following lake pairs (See Fig 1 for locations):

- *Gull Lake and Pokegama Lake*.
  Gull Lake is heavily infested with zebra mussels, with the first found in 1990. The water is very clear. Mussel shells litter the beaches. At the time of the survey, Pokegama Lake (referred to as Lake Pokegama locally) was connected to a water body that is infested with zebra mussels, but no zebra mussels had been found in its waters (however, it was listed as infested later in the year). No additional invasive species have been noted at either lake. Both lakes have similar recreational activities and are popular vacation locations.
- *Lake Koronis and Lake Minnewaska*.
  The first lake in Minnesota found to host starry stonewort (in 2013), Lake Koronis is heavily infested with starry stonewort, but has no other AIS. The starry stonewort fills the water column in large patches of Koronis. Lake Minnewaska has a small infestation of starry stonewort, isolated to a marina (the survey locations were not located in or by the marina). Minnewaska also hosts zebra mussels and Eurasian watermilfoil, *Myriophyllum spicatum L*., a rapidly growing aquatic plant that forms dense mats at the water’s surface. Both lakes have similar recreational activities and are considered more “local” lakes, which are not as popular for vacationing as Gull and Pokegama.

## 4 Survey design and statistical model

### 4.1 Survey design and administration

The survey protocol was developed using standard survey methodology [21, 22], and implemented via Qualtrics. Surveys were administered on tablets using Qualtrics at public lake access points during June, July, and August, 2019. As survey locations included boat ramps, many respondents were using boats, though this was not a requirement for survey participation. Each lake was surveyed for a total of 140 hours, spread out over four five-day periods, spanning weekends and weekdays. All lakes had multiple public lake access points (see Fig 1) —survey locations were rotated. Survey time blocks (morning, afternoon, evening) were also rotated.

Survey administrators (undergraduates) were trained and conducted mock surveys prior to collecting data. During surveying periods, administrators wore matching University of Minnesota hats, shirts, and nametags. They also put up University branded signs saying “Your opinion needed.” Working in twos, they approached recreationists, many of whom were either launching or loading a boat. They identified themselves as University of Minnesota students collecting data, and asked if the recreationists would participate in a voluntary survey about aquatic invasive species. Estimated time of completion, 7 to 10 minutes, was provided (actual median response time was measured as 7.8 minutes). Respondents were asked if they were over 18 before they were allowed to proceed. Respondents were then handed the tablets. Occasionally, administrators were asked to read the survey questions. If so, the administrators complied. Administrators were instructed not to answer any questions except for clarifying ones (e.g., if a respondent did not understand a word used in a question). The questionnaire and the administration protocol were reviewed by the University’s Institutional Review Board.

### 4.2 Survey measures

The survey collected data on willingness to pay to access the lake (see Section 4.1 below), as well as data on multiple variables that could potentially impact willingness to pay, including respondents’ visit (e.g., length of visit, activities), perceived AIS risk, knowledge about AIS, AIS awareness of problem, socio-demographics (e.g., gender, income, education), and travel patterns (e.g., whether they were coming from/returning to home [i.e. whether they were locals]).

#### Recreational activities

The questionnaire included two questions about respondents’ current recreational activities during the visit. The first question asked participants to report the activities they participated in during the visit (e.g., fishing, boating, hiking, socializing, swimming). A follow up question asked respondents to identify the activity that was the primary reason for their visit.

#### Visit motivation

Recreationists’ motivation to visit the lake was measured using eight items [23, 24]. People’s motivation to participate in an activity (e.g., visiting a lake) provides an explanation for why people engage in that activity [23]. In the context of recreation and leisure science, visit motivation has been linked to intention to revisit tourist destination [25], and satisfaction with tourist or visit experience [26, 27]. Respondents were provided with a list of possible reasons why people visit lakes: “to be close to nature”, “to be physically active”, “to be on my own”, “to socialize”, “to view the scenery”, “to get away from the usual demands of life”, “to relax”, and “to experience silence and quiet”. They were asked to rate how important each of the reasons were to them on a five point scale from “not at all important (0)” to “extremely important (4)”. Past work has identified several domains of visit motivation including autonomy (e.g., “to be on my own), nature enjoyment (e.g., to view the scenery, to be close to nature), health/physical rest (e.g., to relax), solitude (e.g, to experience silent and quiet), escape from personal/social pressures (e.g., to get away from usual demands of life), and social motivations (e.g., to socialize) [23, 24].

#### Perceived AIS risk

Perceived risk of AIS was measured using six items. Respondents were asked to rate the extent to which they believe AIS is a risk to various water-related ecosystem services: “habitat for native fish and aquatic plants”, “quality of recreational opportunities (e.g., boating, fishing)”, navigability of waterways”, “economic viability of recreation and tourism businesses”, “cost of water treatment”, and “water quality in Minnesota’s lakes, rivers, and streams”. Response was on a five-point scale from “no risk at all (0)” to “extreme risk (4)”.

#### Awareness of AIS problem

One item was used to measure awareness of AIS problem. Respondents were asked to rate the extent to which they believe AIS are a problem in Minnesota on a four-point scale from “not a problem at all (1)” to “severe problem (4)”.

#### Knowledge about AIS

As the infestation type and magnitude of the particular lake was of importance, we asked the respondents general questions about AIS, including with what AIS they were familiar. We included photographs and general information about three invasive species of concern in Minnesota: Eurasian watermilfoil, zebra mussels, and starry stonewort (S1 Appendix question). We also asked respondents to identify the invasive species that were present in the lake.

The invasive species in the response list, as well as the photos and descriptions, were the same as in the familiarity question listed above, and remained constant across the four lakes. A fourth option, “None of these species are in <lake name>” was also included. While the respondents were required to advance linearly through the survey, there is no guarantee they read the entire question. As such, we designed the survey with a degree of repetitiveness to give the respondents the best chance to retain information regarding the species.

### 4.3 Willingness to pay

#### Survey design

**Fig 2:**
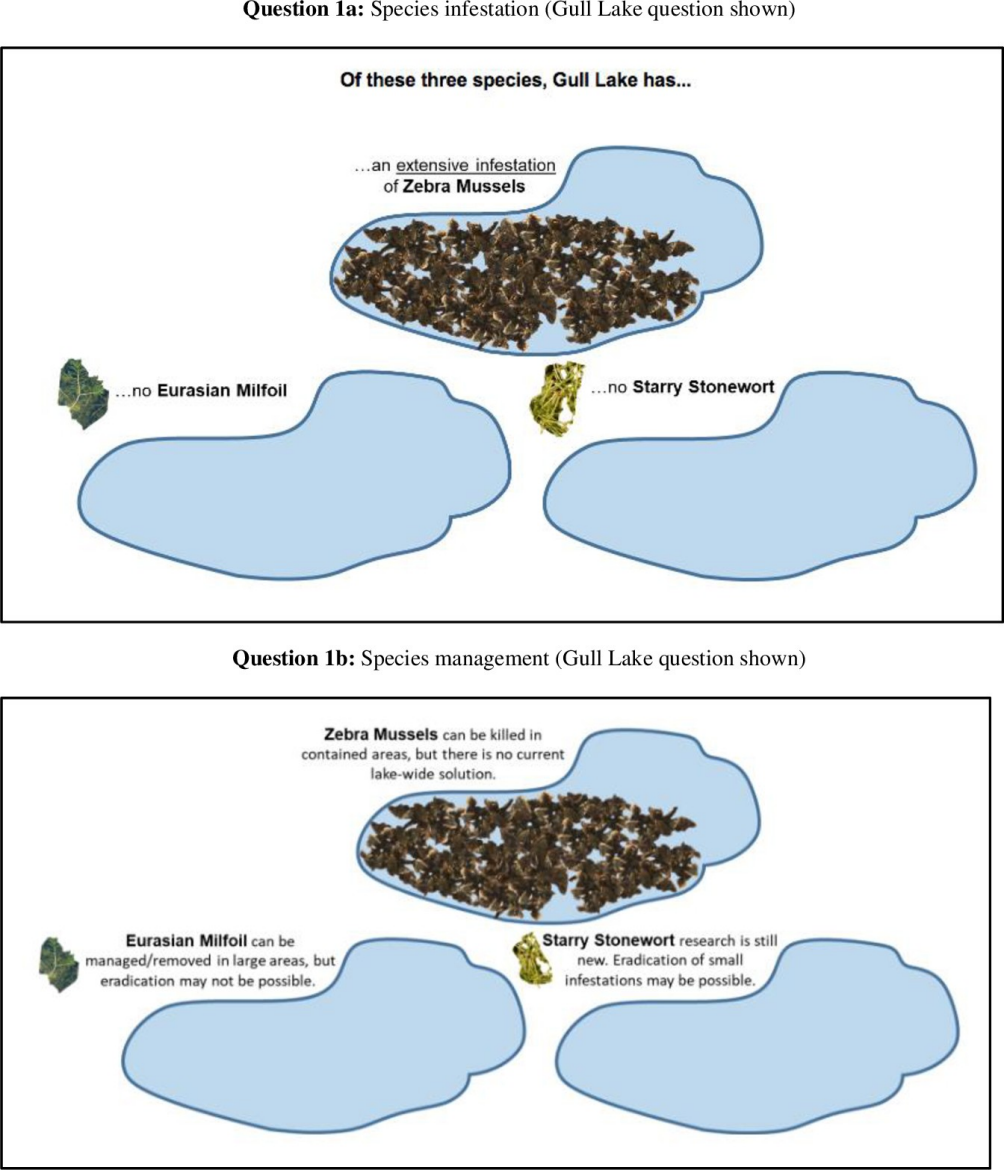
Question 1: Lake infestation.

Willingness to pay questions were designed using recent stated preference guidance [28, 29]. First, respondents were provided with lake specific AIS information (and could no longer back track in the survey to avoid correcting themselves) in a graphical form which emphasized the infestation magnitude (Fig 2, Question 1a). Respondents were then provided information regarding management strategies and potential impact of management, while still emphasizing infestation magnitude (Fig 2, Question 1b), to ensure respondents had information on what is currently possible for AIS management.

**Fig 3:**
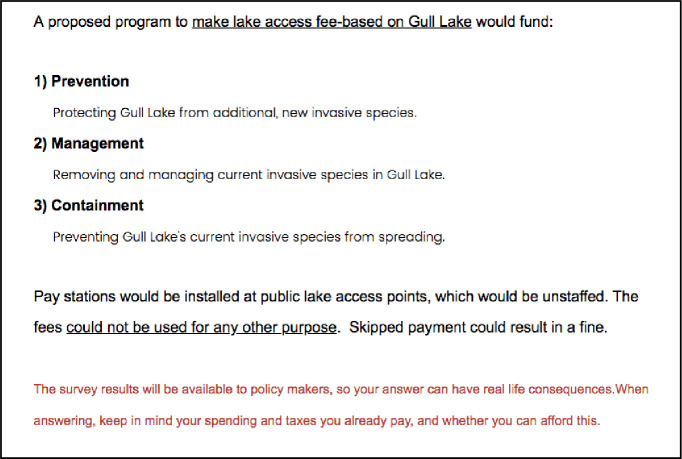
Question 2: Proposed program.

Next, they were provided information on current statewide AIS spending including a comparison to other types of state spending (S2 Appendix question). They were also given information on a proposed program to make lake access fee-based (Fig 3, Question 2). This program stated that funds from making the lake access fee-based would go towards prevention, management, and containment at the individual lake via unstaffed pay stations. Failure to pay could result in a fine. Respondents were also warned that the results would be available to policy makers and that they should keep in mind their spending and taxes, and whether or not they could afford the fees. All this information is important for the collection of willingness to pay data, else respondents are more likely to answer unrealistically, potentially skewing results [32].

And, finally, respondents were asked if they would support a daily fee to access the lake in question (S3 Appendix question) following a double-bounded dichotomous choice format [30]. The dollar values were random—at first the values ranged from $8 to $14. If the respondent answered “No”, they were asked again, with a lower value—between $1 and $7. If they answered, “Yes”, they were asked a higher value—between $15 and $21. Values were chosen with consultation from the Department of Natural Resources.

#### Model

The individual *i*’s willingness to pay, *WTP*_*i*_ is:

$${WTP}_{i}=x_{i}\beta +\varepsilon_{i}$$ (1)

where ***x***_***i***_ is a vector of explanatory variables, with their coinciding coefficients, ***β***. *ε* is the normally distributed error term. Explanatory variables include individual lake, socio-demographics, and measures described in Section 4.2.

The double-bounded question format results in four possible cases, with each case having a different probability of occurring. This is essentially a modified probit model (for full derivations, see [31]), which assumes the error is normally distributed. This is required by the model, as the probability estimates rely upon the cumulative distribution function. Φ. Maximum likelihood estimation (in this case the doubleb command in Stata) is used to estimate model parameters, $\hat{\beta }$, and $\hat{\sigma }$, standard deviation.

Let $v_{i}^{1}$ be the first value seen by respondent *i*, and $v_{i}^{2}$ be the second. The probability that respondent *i* will answer “Yes” to both questions is:

$$\begin{array}{l} \mathrm{Prob}\;(\mathrm{Yes};Yes)=\mathrm{Prob}\left[WTP_{i}\geq v_{i}^{2}\right]\\ \;=1-\Phi \left[\frac{v_{i}^{2}-x_{i}\beta }{\sigma }\right] \end{array}$$ (2)

The probability that respondent *i* will answer “Yes” to the first question, and “No” to the second:

$$\begin{array}{l} \mathrm{Prob}\;(\mathrm{Yes};No)=\mathrm{Prob}\left[v_{i}^{1}\leq WTP_{i}<v_{i}^{2}\right]\\ \;=\Phi \left[\frac{v_{i}^{2}-x_{i}\beta }{\sigma }\right]-\Phi \left[\frac{v_{i}^{1}-x_{i}\beta }{\sigma }\right] \end{array}$$ (3)

The probability that respondent *i* will answer “No” to the first question, and “Yes” to the second:

$$\begin{array}{l} \mathrm{Prob}\;(No;Yes)=\mathrm{Prob}\left[v_{i}^{2}\leq WTP_{i}<v_{i}^{1}\right]\\ \;=\Phi \left[\frac{v_{i}^{1}-x_{i}\beta }{\sigma }\right]-\Phi \left[\frac{v_{i}^{2}-x_{i}\beta }{\sigma }\right] \end{array}$$ (4)

And finally, the probability that respondent *i* will answer “No” to both is:

$$\begin{array}{l} \mathrm{Prob}\;(No;No)=\mathrm{Prob}\left[WTP_{i}<v_{i}^{2}\right]\\ \;=\Phi \left[\frac{v_{i}^{2}-x_{i}\beta }{\sigma }\right] \end{array}$$ (5)

## 5 Results

### 5.1 Response summary

We had a total of 994 respondents who fully answered the survey, a response rate (RR) of 60% (Pokegama: n = 190, 64% RR; Gull: n= 273, 56% RR; Minnewaska; n= 350, 59% RR; Koronis: n= 181, 65% RR). The average respondent identified as a white male, held a college degree, and earned more than 70K per year. Responses are consistent with previous DNR surveys and demographics of Minnesota [33–36]. See Table 1 for demographic and qualitative responses.

**Table 1:** Demographics and qualitative responses (n=994). (a). Demographics. (b). Values.

**(a)**							
		**Full Sample**		**Koro**	**Minn**	**Gull**	**Poke**
**Characteristic**	Range	n	%	%	%	%	%
**Gender**	Female	260	29	23	34	76	33
	Male	644	71	77	66	24	67
**Race**	White	822	83	85	83	82	82
	Non-white	172	17	15	17	18	18
**Age**	Median	45	-	44	42	48	44
	Minimum	18	-	18	18	19	18
	Maximum	90	-	90	85	87	82
**Formal education**	Did not finish high school	0	0	0	0	0	0
	Completed high school	131	15	20	15	12	15
	Some college but no degree	133	15	21	15	15	10
	Associate or vocational degree	176	20	22	22	16	21
	College bachelor’s degree	257	30	24	28	35	30
	Some post-graduate work	55	6	3	8	5	9
	Completed post-graduate degree	117	13	10	11	18	16
**Household income**	Under $20,000	34	5	2	10	1	3
	$20,000-$39,999	69	10	11	12	6	9
	$40,000-$59,999	110	15	17	17	12	15
	$60,000-$79,999	98	14	14	15	10	15
	$80,000-$99,999	96	13	16	11	16	11
	$100,000-$149,999	148	21	22	19	19	23
	$150,000 or more	167	23	17	17	36	23
**Visitation Frequency**	Several times a week	225	26	29	29	17	31
	Once a week	88	10	10	9	9	14
	Several times a month	166	19	16	21	18	20
	Once a month	69	8	5	10	8	7
	Several times a summer	152	18	25	13	19	19
	Once during the summer	121	14	9	13	22	9
	Every few summers	43	5	5	6	8	0
**Residence**^1^	Local	454	60	67	63	42	72
	Non-Local	307	40	33	37	58	18
**Primary Visitation Purpose**	Fishing	419	43	75	32	41	35
	Boating	209	21	11	16	29	31
	Watersports	95	10	3	9	14	12
	Swimming	87	9	3	16	3	10
	Socializing	50	5	2	8	56	3
	Relaxing	44	5	2	8	2	4
	Hiking	16	2	2	3	1	0
	Picnicking	8	1	1	2	0	1
	Art	8	1	1	2	0	0
	Other	39	4	2	5	4	5
**(b)**							
**Characteristic**	**Range**	**n**	%				
**AIS Knowledge**	Correct	279	31	43	26	34	25
	Incorrect	616	69	57	74	66	75
**Awareness of AIS Problem**	Not a problem at all	32	3	2	3	5	3
	Slight Problem	107	11	14	12	8	10
	Moderate Problem	398	40	40	40	40	41
	Severe Problem	413	42	43	41	43	40
	Don’t know/Not sure	39	4	2	4	4	5
**Perceived AIS Risk**^2^	Median	Moderate/ High Risk		Mod/ High Risk	Mod/ High Risk	Mod/ High Risk	Mod/ High Risk
**Visit Motivation “To be on my own”**^3^	Median	Moderately Important		Mod Import	Mod Import	Mod Import	Mod Import

^1^ Locals were respondents who indicated they lived at the lake, were the lake for a day trip and were coming from their primary residence, or were staying at the lake for multiple nights but were staying at home.

^2^AIS Risk is represented by the sum across the risk categories provided in Section 3, where the options are represented as ^2^ Perceived AIS Risk is represented by the sum across the risk categories provided in Section 3, where the options are represented as scalar variables (0: No risk at all, 1: Slight risk, 2: Moderate risk, 3: High Risk, 4: Extreme Risk). The median response was 2.7, or between moderate and high risk.

^3^ Eight visitation reasons were provided (see Section 3), one of which was “To be on my own”, which represented as scalar variables (0: Not at all important, 1: Slightly important, 2: Moderately important, 3: Very important, 4: Extremely important). The median response was 2, or moderately important.

There are a number of differences between the lakes; in addition to the information in Table 2, summary statistics for all included variables are provided in S2 Appendix table, grouped by lake. Awareness of problem is fairly consistent across all four lakes, and is not significantly different between any lakes. Knowledge of AIS is highest at Lake Koronis, significantly different from all other lakes. Koronis is very well known for having starry stonewort. Gull is relatively well known for its zebra mussel infestation, but it is frequented by non-locals who may not be as familiar (being a local was correlated with being correct about the AIS in the lake). Pokegama has no AIS; Minnewaska had three, and neither are well known for their AIS. Perceived Risk is highest at Koronis, lowest at Minnewaska (significant at 95% level). Again, the higher perceived risk at Koronis may be due to the well-known nature of the starry stonewort infestation. Koronis was also interesting because it had far more people who indicated that fishing was their primary reason for visiting—about double that of any other lake. It also had the highest score for “to be on my own” visit motivation, which is correlated with fishing being the primary purpose. There were a number of people kayaking and fishing at Koronis, which may have helped generate these higher numbers. Pokegama has the highest percent of locals, which could be influencing why its estimated willingness to pay scores are on the lower end (even if not significantly). Gull has the lowest percent of locals, but high income and high education.

**Table 2:** Willingness to pay model (n =538; Wald = 656)^1^.

Coefficient	Variable Description	Mean	Std. Error	P Value
$\hat{\beta_{1}}$	*Initial Value* ($v_{i}^{1}$)	0.2901**	0.1472	0.049
$\hat{\beta_{2}}$	*Awareness of AIS problem*	1.9109*	0.6697	0.004
$\hat{\beta_{3}}$	North * Awareness of AIS problem	-0.7187**	0.3389	0.034
$\hat{\beta_{4}}$	*Perceived AIS risk*	0.2025**	0.0963	0.035
$\hat{\beta_{5}}$	*Visit motivation*	-1.0045*	0.3195	0.002
$\hat{\beta_{6}}$	*Local*	-2.2465*	0.8464	0.008
$\hat{\beta_{4}}$	*Education*	0.3588	0.2505	0.152
$\hat{\beta_{5}}$	*Gender*	1.6922	0.9021	0.061
$\hat{\beta_{6}}$	*Aged 45 or Greater*	1.8544**	0.8545	0.030

*indicates significance at the 99% confidence level.

**indicated significance at the 95% confidence level

^1^ *Gender* (1:Female; 0:Male), *Local* (1: respondents indicated they lived at the lake, were the lake for a day trip and were coming from their primary residence, or were staying at the lake for multiple nights but were staying at home; 0: respondents indicated otherwise), *Gender* (1: female; 0: male); no respondents choose non-binary, *Aged 45 or greater* (1: respondent is 45 years of age or older; 0: respondent is less than 45), and *North* (1: Gull or Pokegam; 0:Minnewaska or Koronis);, *Initial Value* is the first bid offered in the WTP question. Scalar variables are *Awareness of AIS Problem* (See S1 Appendix question) (1: Not a problem at all, 2: Slight Problem, 3: Moderate Problem, 4: Severe Problem), *Perceived AIS Risk* is the sum across the risk categories, where the options are (0: No risk at all, 1: Slight risk, 2: Moderate risk, 3: High Risk, 4: Extreme Risk), *Visit Motivation* measured how important it was for the respondent to “be on my own” (0: Not at all important, 1: Slightly important, 2: Moderately important, 3: Very important, 4: Extremely important), and *Education* (1: Did not complete high school, 2: Completed high school, 3: Some college but no degree, 4:Associate degree or vocational degree, 5: College bachelor’s degree, 6:Some postgraduate work but no degree, 7: Completed graduate degree).

**Fig 4:**
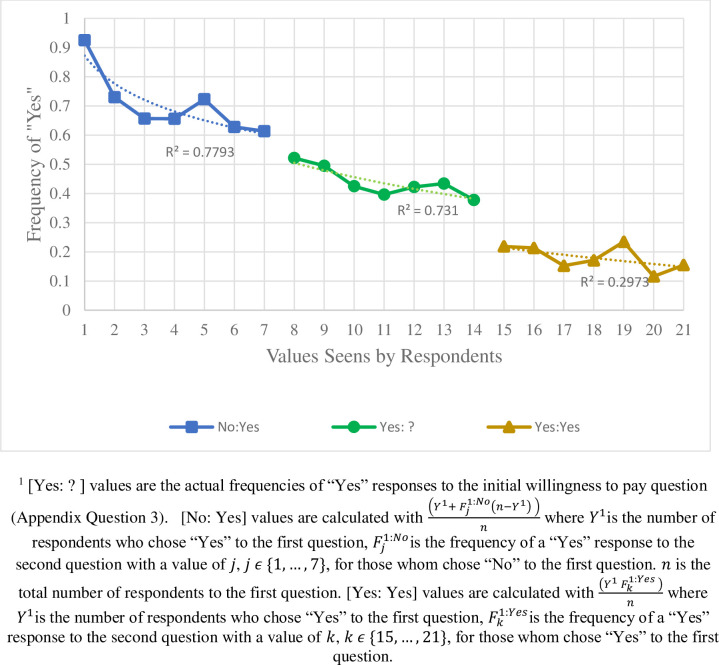
Calculated frequency of “Yes” responses to willingness to pay questions by value amount, $v_{i}^{1}\;and\;v_{i}^{2}$ (overall R^2^ = 0.937)^1^.

To check that the willing to pay responses (S3 Appendix question) followed a downward sloping demand curve—i.e. that fewer people are willing to pay higher amounts, we estimated the frequency of “Yes” responses for each value offered. The initial values are given (random values from $8 to $14). Those who answered “No” to the first question were provided with lower values in the second question (random values from $1 to $7), and those who answered “Yes” to the first question, and were provided with higher values in the second question (random values from $15 to $21). “Yes” response frequency to the initial willingness to pay question were lower for higher values, ranging from upper 30%s to lower 50%s, with a statistically significant negative slope (Fig 4). To estimate the frequencies of the responses to the second values, we assumed that people who answered “Yes” to the first set would also answer “Yes” to the lower values and people who answered “No” to the first set would answer “No” to the higher values.

The chart shows a clearly negative relationship between frequencies of “Yes” response and valued proffered. There are noticeable gaps between the sets of values, which are likely evidence of response biases. People who answered “No” initially may be experienced some guilt or pressure to answer “Yes” when faced with a lower value, which may increase the frequency—higher levels of affirmative responses can be a result of a multi-question format [32]. However, people who answered “Yes” initially may be experiencing some annoyance at being asked a higher value, which may decrease the frequencies. Still, we do not see extremely high response rates at the highest levels, which can be an issue in contingent valuation work [37].

Protest votes, which are willingness to pay values of zero given due to reasons beyond an actual value of zero (i.e. philosophical arguments), were excluded from the estimation of willingness to pay and were determined by a follow-up question to those choosing “No” to both valuation questions. Respondents were given the following options: “I already pay enough in taxes/fees for aquatic invasive species”, “I would support more taxes/fees for aquatic invasive species in the whole state, but not for <lake name> alone”, I will not support making <lake name> public lake access fee-based, regardless of the amount or what the fee is for”, “I would support a fee, but this amount is too expensive/I cannot afford this much”, “I do not believe we should fight aquatic invasive species”, and “Other: please specify:”. Determining which answers are protest votes and which are true zeros is not an exact science. However, if the answer indicates the respondent has no value for the public good (e.g. “I do not believe we should fight aquatic invasive species”) or cannot afford the fee (e.g. “I would support a fee, but this amount is too expensive/I cannot afford this much”), the answers are considered true zeros [33, 34]. For the write-in answers, we used our best judgment as to whether the responses were protests or not—if they did not write anything in, we considered it a true zero. Protest votes were determined to make up about one-half of people who chose “No” to both questions, 23% of the total sample.

### 5.2 Willingness to pay estimation

We estimated mean willingness to pay for the total viable sample (n = 705) with a simple model that included no explanatory variables (Eq 1 with a constant and no vector of variables); the result was $9.37. We also estimated the willingness to pay for each lake. The lowest was for Pokegama, $8.15; the highest was for Koronis, $9.80. Interestingly, all values were within each other’s confidence intervals (95%), seemingly suggesting there are fewer differences in willingness to pay between the lakes than we had posited (Table 3). We next calculated that the mean willingness to pay as $9.87 by estimating the parameters in Eq 1 (Table 2), which is 50 cents higher than in our simple model--for a discussion of non-response bias, see Section 5.3. Our preferred model included initial value offered (significant at the 95% level), perceived risk of AIS (significant at 95% level), magnitude of desire to “be on your own” (significant at 99% level), whether a respondent is a local (significant at 99% level), education (significant at 85% level), whether a respondent identifies as female (significant at 90% level), whether a respondent is aged 45 years or older (significant at the 95% level), AIS awareness of problem (significant at 99% level), and an interaction variable between AIS awareness of problem and whether the lake is one of the northern pair (Pokegama or Gull) (significant at the 95% level)

**Table 3:** Comparison of WTP estimates from simple model and preferred model.

		Simple Model			Preferred Model	
	n	WTP	CI (95%)	n	WTP	CI (95%)
All	705	$9.37	$8.60 to $10.13	538	$9.87	$9.07 to $10.67
Koronis	124	$9.80	$7.94 to 11.66	102	$10.40	$9.28 to 11.51
Minnewaska	244	$9.64	$8.42 to $10.85	185	$10.34	$9.27 to 11.40
Pokegama	142	$8.15	$6.09 to $10.22	103	$8.96	$7.77 to 10.14
Gull	195	$9.37	$8.09 to $10.89	148	$9.56	$8.38 to 10.74

We compared our preferred model (Table 2) with alternative models that included the different lakes, alternate primary purposes of visit (fishing was predictive but the others were not), alternate motivation categories (the other options were not predictive as compared to “to be on my own”), alternate risk categories, and demographics. Alternative models did not result in discordant results or interpretation. Two alternative models are presented in S1 Appendix table, which provides similar willingness to pay results. These models were selected to show lake variables and three variables that were predictive in certain combinations: income, AIS Knowledge, and fishing as a primary visit purpose. Models were compared in terms of statistical fit (Wald’s tests, Akaike’s / Bayesian information criteria) and the estimated significance of impact on WTP. Our preferred model was chosen based on overall fit, significance of variables, correlation of variables (See S3 Appendix table) and sample size. We also tested our methodology by looking at classification error for a test set (80/20 split) and found good agreement (not shown).

Initial value presented impacted willingness to pay, by about 30 cents for each dollar increase in initial offer, evidence that anchoring may be occurring, which is common with this format of questioning [38]. Anchoring is a phenomenon where people base their willingness to pay on the apparent price of an item. As such, a higher initial value may increase respondents’ final willingness to pay. Locals, people who said they were staying at/returning to home were willing to pay $2.25 less than people on trips. This may be because people who live at the lake feel they should not pay visitation fees or because they visit with more frequency--the fee proposed was a daily fee, not a yearly. Visitation frequency was significantly different between the two groups. Locals visited an average of several times a month, whereas as others visited once a month on average—however inclusion of both visitation variables did not increase model fit, nor did solely including visitation frequency (not shown).

Education was not very significant (P = 0.16), but showed the expected sign—where more educated people are willing to pay more. Education is correlated with income (S3 Appendix table), which we discuss more below, but feel is important. Gender was more significant (P=0.06), indicating that people who identify as women are willing to pay quite a bit more--$1.69. Being a woman was correlated somewhat with risk perception, which has been found before [39]. Adams et al [40] found that demographic variables changed willingness to pay by about |$1| for invasive plant control. Nunes and Van der Bergh [41] found very little impact of socio-demographics on willingness to pay for harmful algal bloom prevention; Chakir et al. [42] found the same for socio-demographics impact on willingness to pay to reduce negative impacts of an invasive beetle.

Perceived AIS risk, AIS awareness of problem, and desire to “be on your own”, however, were significant. Risk was represented as a sum of equivalent risk responses across five categories. Extrapolating with our model results, the person who perceived AIS the most risky would be willing to pay about $4.86 more than the person who perceived the least risk. Problem awareness impact was large (and significant at 99%). Someone who viewed AIS as a severe problem would be willing to pay about $5.73 more than someone who viewed AIS as not a problem, if they were at Koronis or Minnewaska. If they were at Gull or Pokegama, this impact is lower--$3.58. This may have to do with the type of lake—both Pokegama and Gull are clearer lakes, viewed as recreation destinations. Minnewaska and Koronis are more known for having water quality impacts. Perhaps even if people at the northern lakes feel that AIS in Minnesota are a problem, their willingness to pay is lower at a lake they view as less impacted by AIS. Someone who ranked “to be on your own” as “extremely important” was willing to pay $4.02 less than someone who ranked “to be on your own” as “not at all important”. AIS awareness of problem is less correlated with other variables than perceived AIS risk; the two are quite correlated with each other (S3 Appendix table). We chose to use still use both since they measure different social-psychological constructs.

It is interesting that we did not find more significance related to the individual lakes. What is apparent is that individual respondents’ characteristics may be more important than which AIS is present or magnitude of infestation.

### 5.3 Problematic variables and non-response bias

We did not include income in our preferred model because a third of respondents refused to provide this information. A number of demographics are correlated with income, including education and whether a respondent is a local. Additionally, including income did not result in a better overall model. Though, we did include it in alternative models (S2 Appendix table), and it behaved as expected-- Income increased willingness to pay—the difference between the smallest income bracket (up to $20,000) and the largest (over $200,000) equated to about $2.78. We also did not include AIS Knowledge in our preferred model. While AIS Knowledge was associated with higher willingness to pay in some model formulations (see S2 Appendix table), its inclusion reduced overall model fit. We felt it was problematic due to the questions being “harder” at some lakes (i.e. at Minnewaska you had to select three to be correct; at Pokegama, the correct answer was none, but zebra mussels were found there after the survey).

We took a look at the people who answered the income question vs. the people who did not, as we thought it may tell us something about non-response bias. Those who did not answer did not differ vary significantly from those who did in any of the variables tested, except for in education. We added a binary variable to our preferred model to account for answering the income question or not, and found that answering the question was associated with $2.85 more in willingness to pay (significant at 99% level [not shown]), which is about the magnitude of the income impact from the lowest to highest income category. It is quite possible that people who do not answer for income have lower income, which jives with the lower education level.

We also examined time to complete the survey, which averaged less than 8 minutes. While overall time was not significant in willingness to pay [not shown], those who were in the lower quartile (less than 6 min) were willing to pay about $1.70 less, with the significance at the 90% level (not shown). People who did not answer the income question were more likely to be in this lower quartile, but their mean time to completion was not significantly different that the remaining respondents. Still, this may point at a form of non-response bias. Additional evidence for potential non-response bias lies in the results of willingness to pay estimates on different subsamples. The simplest model uses the entire sample, and gives us slightly lower WTP score than the preferred sample (Table 3), while these may not be significantly lower than the preferred model, it made us curious about the one-quarter of the respondents in the usable sample who were not included in the preferred. These respondents had not responded to all included questions, so there is overlap with the folks who did not answer the income question. We used the simple model on this “non-responder” group and found that their willingness to pay was indeed lower (S4 Appendix table), $7.14. All of this implies that there could certainly be a concern over non-response.

## 6 Discussion and conclusion

We assessed recreationists’ willingness to pay an access fee for AIS management, prevention, and containment in four Minnesota lakes: Gull, Pokegama, Koronis, and Minnewaska. The four chosen lakes were in two pairs with each pair chosen to be as similar as possible with the exception of their aquatic invasive species (AIS) infestation magnitude. The northern pair was Gull and Pokegama. Gull is host to an extensive zebra mussel, *Dreissena polymorpha*, population. Pokegama was considered free of infestation as of the time of the survey, however was listed as infested with zebra mussels later in the year. The southern pair was Koronis and Minnewaska, where Koronis was one of the first lakes in the state infested with starry stonewort, *Nitellopsis obtuse*, and has an extensive population. Minnewaska has a minor, contained starry stonewort population, but also has Eurasian watermilfoil, *Myriophyllum spicatum L*, and zebra mussels.

We found that respondents are willing to pay $9 to $10 daily to access these lakes, a not unsubstantial sum. We did not find that presence at infested lakes versus uninfested/less infested lakes affected willingness to pay—in other words, we could not detect willingness to pay differences within the lake pairs once we had accounted for other variables. We did find that certain demographic, social-psychological, and travel-related variables were predictive.

Locals were willing to pay less than visitors to the region. This could be related to frequency of visitation, which was higher for locals. A daily use fee could result in a significant amount over the course of a season with high visitation rates. While we did not include income in our preferred model due to sampling issues, it was predictive of higher willingness to pay values in alternate models. Education and gender were not highly significant, but each had the expected sign. Those over the age of 45 were willing to pay more, as well.

Demographic and descriptive variables provide information about *who* is willing to pay, but not about *why* they are willing to pay. We found that social-psychological variables: awareness of AIS problem, perceived risk, and visit motivation, were important determinants of willingness to pay for AIS management.

Our findings suggest that people who believe to a greater extent that AIS are a problem are more willing to pay for AIS management. The norm activation theory [3] suggests that awareness of a problem is an important first step in a cognitive process that leads to action. Thus, people who believe AIS is a problem are more likely to take action (i.e., willingness to pay). Interestingly, we also found that the influence of awareness of AIS Problem was stronger at the southern lake pair than the northern lake pair. The northern lakes are more clear and generally seen as “nicer” than the southern lakes. It is possible the even though the measurement of general awareness of AIS problem (the question was not directed specifically to the lake in question) did not differ between lakes, being at a lake that seemed “nicer” might reduce the willingness to pay.

We also found that recreationists who perceive greater risk of AIS to ecosystem services are willing to pay more for AIS management. Past work on risk perception, particularly around climate change beliefs and actions, indicate that perceptions of risk are an important predictor of public willingness to take actions to address climate change [16, 43]. Our findings provide support for the link between risk perception and environmental action in the context of invasive species management.

Finally, we found that recreationists with a privacy or autonomy motive [28] (i.e., desire to be on their own) are willing to pay less. Past work has found that the autonomy motive is related to affective attachment (i.e., emotional bond with a place or setting), as well as place identity (i.e., a place or setting becoming part of one’s identity) [28]. This means that people who value autonomy and privacy generate strong sense of emotional connection with a physical space (e.g., city, lake). In our study, it is possible that people with an autonomy motive may have developed strong emotional connection to the lake, strong enough where they feel they should not have to pay to access the space they have an emotional connection with. However, since we did not measure attachment or place identity, the links among autonomy motive, attachment, and behavior is unclear and provides an interesting area for future research.

From a management perspective, our findings suggest that strategies that highlight the extent of the AIS problem, and draw links between AIS and their risks to ecosystem services may be successful in garnering more support for AIS management, regardless of local lake infestations. While these strategies can help garner public support for AIS management (through their willingness to contribute money to a hypothetical market), policy makers and resource managers should be cognizant of any resistance to the payment vehicle, in this case, a public access fee / parking fee. Public access to lakes in Minnesota is currently free. Protest votes from almost a quarter of our sample indicate that a substantial proportion of recreationists may be opposed to access-based fees. Additionally, like many willingness to pay studies, non-response bias may be a concern. Public acceptability of public access fees, and other policy options is a promising area for future research.

AIS are a growing and ecologically complicated problem worldwide, with substantial support for management from the public. Perhaps surprisingly, we highlight that AIS species and infestation levels may not always be predictive of willingness to pay for management, even when the proposed fees are lake specific. However, we show that recreationists’ willingness to pay *is* influenced by their beliefs about whether AIS is a problem, their perceptions of risks associated with AIS, and motivations for visiting a lake, providing support for the inclusion of social-psychological variables in willingness to pay models, and in AIS management discussions.

## Supporting information

S1 Appendix question AIS awareness. (TIF)

S2 Appendix question Current spending. (TIF)

S3 Appendix question Willingness to accept (Gull Lake version). (TIF)

S1 Appendix table Preferred model vs. alternate models^1^. (DOCX)

S2 Appendix table Comparison of explanatory variables by lake^1^. (DOCX)

S3 Appendix table Variable correlation matrix (Spearman’s). (DOCX)

S4 Appendix table Estimated willingness to pay for responders in preferred model and non-responders. (DOCX)
