# Supplementary figures and images for: Recreationist willingness to pay for aquatic invasive species management
Source: PLoS One. 2021 Apr 14;16(4):e0246860. doi: 10.1371/journal.pone.0246860 (PMC8046257; doi:10.1371/journal.pone.0246860)

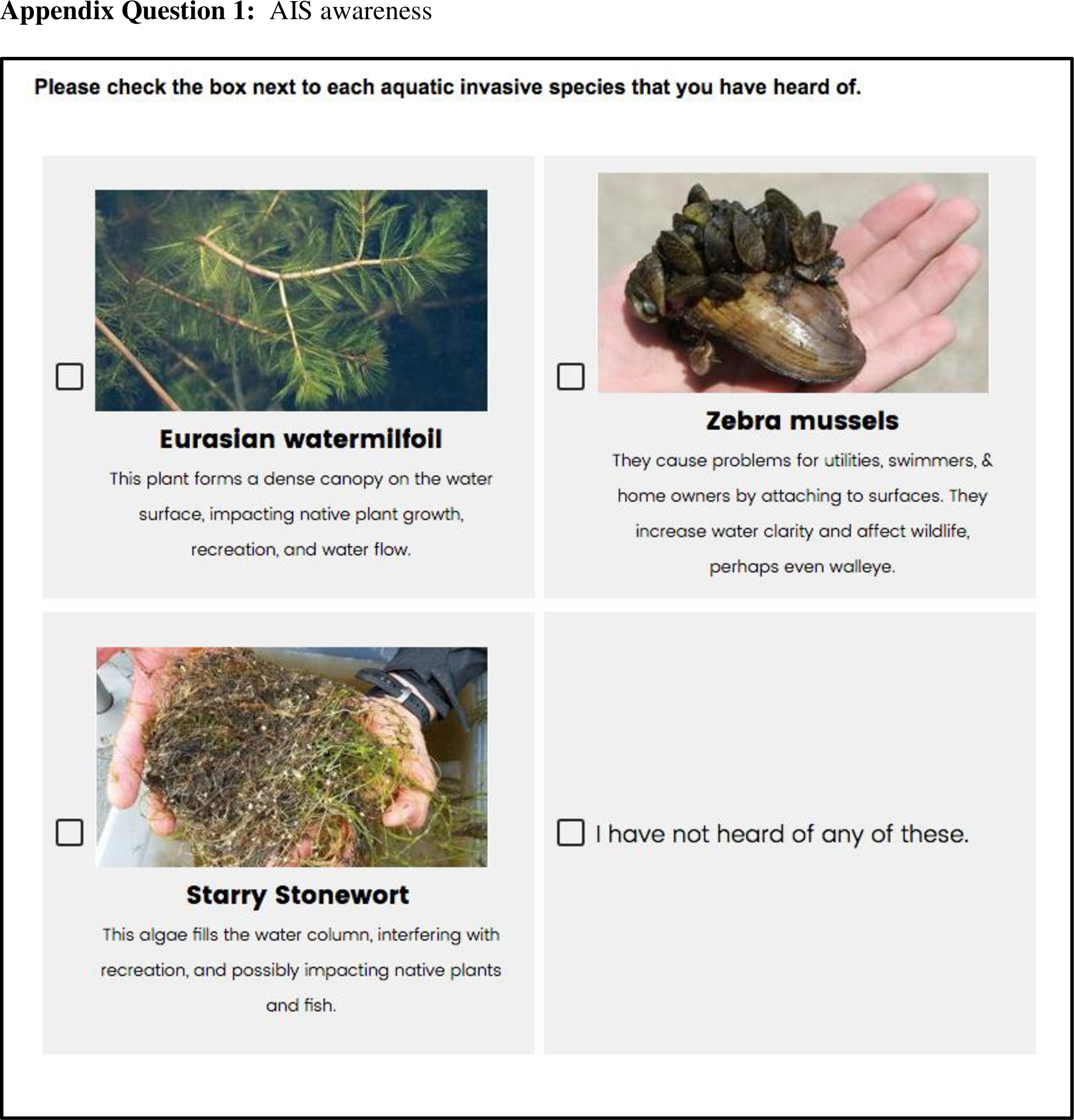

Supplement: S1 Appendix question — (TIF) [file pone.0246860.s001.tif]

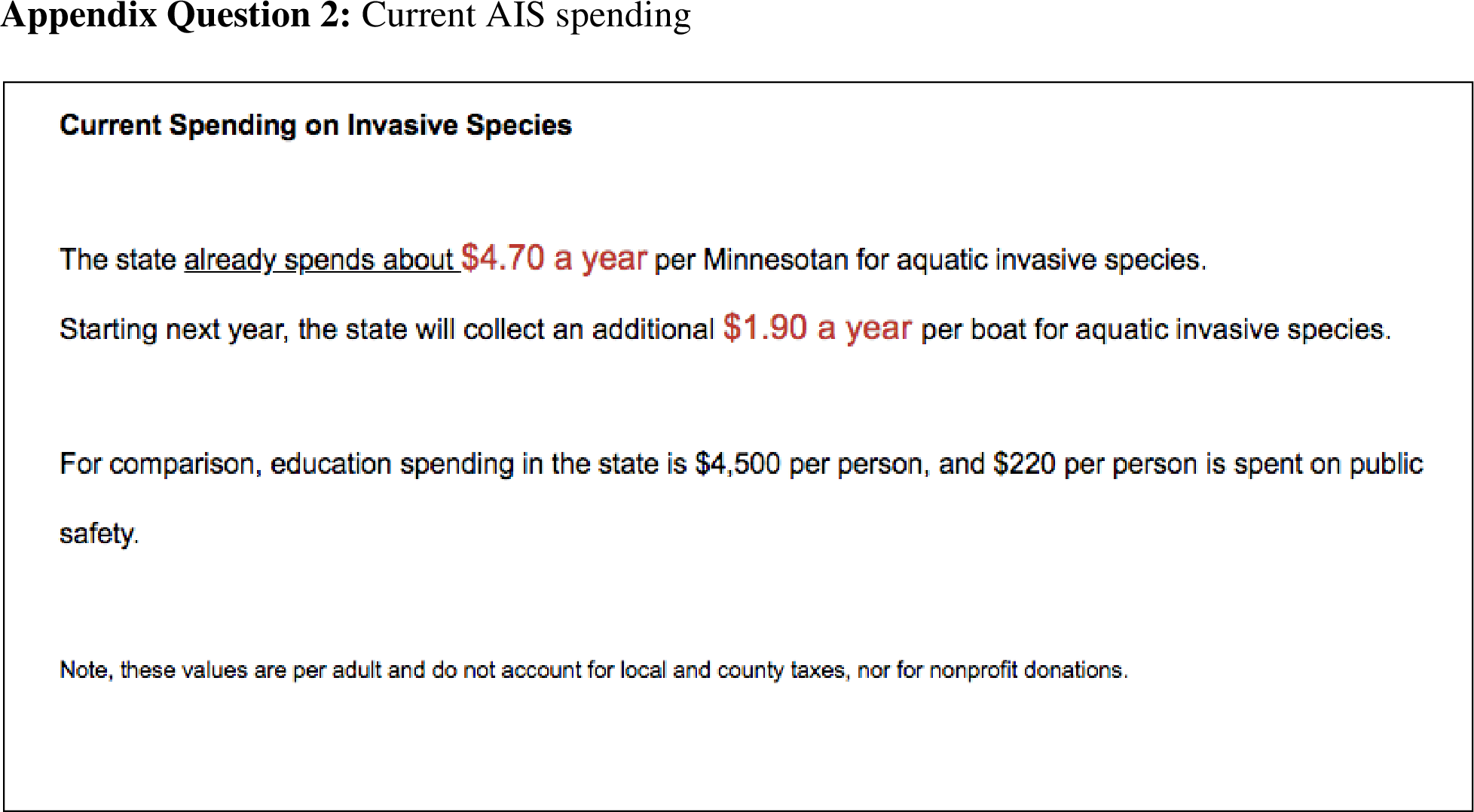

Supplement: S2 Appendix question — (TIF) [file pone.0246860.s002.tif]

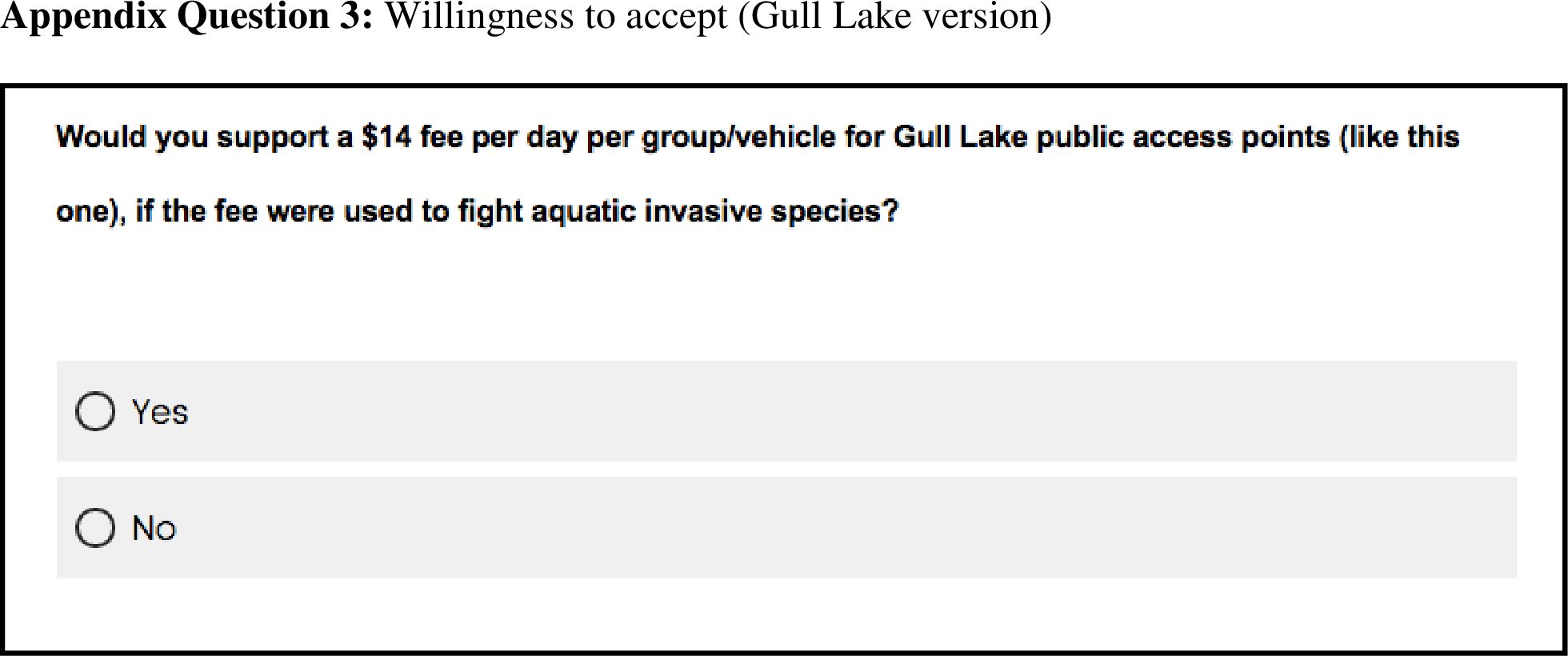

Supplement: S3 Appendix question — (TIF) [file pone.0246860.s003.tif]
