# Supplementary material for: Recreationist willingness to pay for aquatic invasive species management
Source: PLoS One. 2021 Apr 14;16(4):e0246860. doi: 10.1371/journal.pone.0246860 (PMC8046257; doi:10.1371/journal.pone.0246860)
Supplement: S1 Appendix table — (DOCX) [file pone.0246860.s004.docx]

**S1 Appendix table.** Preferred model vs. Alternate Models^1^

| **Coefficient** | **Variable Description** | **Preferred Model** | **Alternate 1** | **Alternate 2** |
| --- | --- | --- | --- | --- |
|  |  | **n = 538;**  **Wald= 656**  **WTP = $9.87** | **n = 462; Wald=617**  **WTP = $10.16** | **n = 553; Wald=635**  **WTP = $9.82** |
| $\hat{\beta_{1}}$ | *Initial Value* ($v_{i}^{1}$) | 0.2901** | 0.403072** | 0.312476** |
| $\hat{\beta_{2}}$ | *Awareness of AIS problem* | 1.9109* | 2.676548* | 2.561663* |
| $\hat{\beta_{3}}$ | *North * Awareness of AIS problem* | -0.7187** | -1.46261 | -0.7013** |
| $\hat{\beta_{4}}$ | *Perceived AIS risk* | 0.2025** | 0.256308** | - |
| $\hat{\beta_{5}}$ | *Visit motivation* | -1.0045* | -1.05662* | - |
| $\hat{\beta_{6}}$ | *Local* | -2.2465* | -2.42802* | -1.96731** |
| $\hat{\beta_{7}}$ | *Education* | 0.3588 | - | 0.290375 |
| $\hat{\beta_{8}}$ | *Gender* | 1.6922 | - | 1.696426 |
| $\hat{\beta_{9}}$ | *Aged 45 or Greater* | 1.8544* | - | 1.0563 |
| $\hat{\beta_{10}}$ | *AIS Knowledge* | - | - | 2.212537** |
| $\hat{\beta_{11}}$ | *Fishing* | - | - | -2.02404** |
| $\hat{\beta_{12}}$ | *Pokegama Lake* | - | - | - |
| $\hat{\beta_{13}}$ | *Gull Lake* | - | 0.390505 | - |
| $\hat{\beta_{14}}$ | *Minnewaska Lake* | - | -2.84054 | - |
| $\hat{\beta_{15}}$ | *Koronis Lake* | - | -2.07544 | - |
| $\hat{\beta_{16}}$ | *Income* | - | 0.252276** | - |

*indicates significance at the 99% confidence level.

**indicated significance at the 95% confidence level

^1^ Indicator variables are *Fishing* (1: Fishing chosen as primary reason for visit; 0: All others*), Gender* (1:Female; 0:Male), *Local* (1: respondents indicated they were staying at/coming from home; 0: respondents indicated they were not), *Gender* (1: female; 0: male); no respondents choose non-binary.  *Aged 45 or greater* (1: respondent is 45 years of age or older; 0: respondent is less than 45), *AIS Knowledge* (1: Correctly identified AIS in the lake; 0: Did not), and *Lake* variable*s* (1: the specified lake; 0: a different lake). *Initial Value* is the first bid offered in the WTP question. Scalar variables are *Income* (1 through 12; 1: Income < $20,000, 2: $20,000 ≤ Income ≤ $29,999, 3: $30,000 ≤ Income ≤ $39,999, 4: $40,000 ≤ Income ≤ $49,999,5: $50,000 ≤ Income ≤ $59,999, 6: $60,000 ≤ Income ≤ $69,999, 7: $70,000 ≤ Income ≤ $79,999, 8: $80,000 ≤ Income ≤ $89,999, 9: $90,000 ≤ Income ≤ $99,999, 10: $100,000 ≤ Income ≤ $149,999, 11: $150,000 ≤ Income ≤ $199,999, 12: Income ≥ $200,000), *Awareness of AIS Problem* (0: Not a problem at all, 1: Slight Problem, 2: Moderate Problem, 3: Severe Problem), *Perceived AIS* *Risk* is the sum across the risk categories, where the options were (0: No risk at all, 1: Slight risk, 2: Moderate risk, 3: High Risk, 4: Extreme Risk), *Visit Motivation* measured how important it was for the respondent to “be on my own” (0: Not at all important, 1: Slightly important, 2: Moderately important, 3: Very important, 4: Extremely important), and *Education* (1: Did not complete high school, 2: Completed high school, 3: Some college but no degree, 4:Associate degree or vocational degree, 5: College bachelor’s degree, 6:Some postgraduate work but no degree, 7: Completed graduate degree).
