# Supplementary material for: Recreationist willingness to pay for aquatic invasive species management
Source: PLoS One. 2021 Apr 14;16(4):e0246860. doi: 10.1371/journal.pone.0246860 (PMC8046257; doi:10.1371/journal.pone.0246860)
Supplement: S2 Appendix table — (DOCX) [file pone.0246860.s005.docx]

**S2 Appendix table.** Comparison of explanatory variables by lake.**^1^**

Table 3(a): Mean values.

|  | Gull | Pokegama | Koronis | Minnewaska |
| --- | --- | --- | --- | --- |
| *AIS Knowledge* | 31 | 24 | 43 | 23 |
| *Perceived AIS Risk* | 15.44 | 15.91 | 16.80 | 15.17 |
| *Awareness of Problem* | 2.39 | 2.26 | 2.39 | 2.28 |
| *Income* | 8.86 | 8.09 | 7.67 | 6.76 |
| *Local* | 42% | 72% | 67% | 63% |
| *Visit Motivation* | 2.09 | 1.94 | 2.25 | 2.00 |
| *Fishing* | 36% | 28% | 70% | 29% |
| *Education* | 4.64 | 4.64 | 4.06 | 4.31 |
| *Gender* | 27% | 34% | 29% | 37% |
| *Aged 45 or Greater* | 69% | 61% | 55% | 55% |

Table 3(b): *AIS Knowledge*: P-values to reject null of equal means

|  | Gull | Pokegama | Koronis |
| --- | --- | --- | --- |
| Pokegama | 0.2385 | - | - |
| Koronis | 0.0217** | 0.0019* | - |
| Minnewaska | 0.0905*** | 0.8364 | 0.0001* |

Table 3(c): *Perceived Risk*: P-values to reject null of equal means

|  | Gull | Pokegama | Koronis |
| --- | --- | --- | --- |
| Pokegama | 0.1230 | - | - |
| Koronis | 0.0246** | 0.1498 | - |
| Minnewaska | 0.5948 | 0.1849 | 0.0046* |

Table 3(d): *Awareness of Problem*: P-values to reject null of equal means

|  | Gull | Pokegama | Koronis |
| --- | --- | --- | --- |
| Pokegama | 0.1378 | - | - |
| Koronis | 0.9515 | 0.1488 | - |
| Minnewaska | 0.1017 | 0.8519 | 0.1230 |

Table 3(e): *Income*: P-values to reject null of equal means

|  | Gull | Pokegama | Koronis |
| --- | --- | --- | --- |
| Pokegama | 0.0489** | - | - |
| Koronis | 0.0041* | 0.3356 | - |
| Minnewaska | <0.0001* | 0.0011* | 0.0381** |

Table 3(f): *Local*: P-values to reject null of equal means

|  | Gull | Pokegama | Koronis |
| --- | --- | --- | --- |
| Pokegama | <0.0001* | - | - |
| Koronis | <0.0001* | 0.9953 | - |
| Minnewaska | 0.0029* | 0.0122* | 0.0285** |

Table 3(g): *Visit Motivation*: P-values to reject null of equal means

|  | Gull | Pokegama | Koronis |
| --- | --- | --- | --- |
| Pokegama | 0.2862 | - | - |
| Koronis | 0.9515 | 0.0538** | - |
| Minnewaska | 0.4888 | 0.6387 | 0.0916** |

Table 3(h): *Fishing*: P-values to reject null of equal means

|  | Gull | Pokegama | Koronis |
| --- | --- | --- | --- |
| Pokegama | 0.1435 | - | - |
| Koronis | <0.0001* | <0.0001* | - |
| Minnewaska | 0.1143 | 0.8977 | <0.0001* |

Table 3(i): *Gende*r: P-values to reject null of equal means

|  | Gull | Pokegama | Koronis |
| --- | --- | --- | --- |
| Pokegama | 0.1428 | - | - |
| Koronis | 0.5465 | 0.0344** | - |
| Minnewaska | 0.4273 | 0.7195 | 0.2080 |

Table 3(j): *Educatio*n: P-values to reject null of equal means

|  | Gull | Pokegama | Koronis |
| --- | --- | --- | --- |
| Pokegama | 0.1522 | - | - |
| Koronis | 0.0566 | 0.0443 | - |
| Minnewaska | 0.0052** | 0.0033* | 0.9233 |

Table 3(k): *Aged 45 or Greater*: P-values to reject null of equal means

|  | Gull | Pokegama | Koronis |
| --- | --- | --- | --- |
| Pokegama | 0.9534 | - | - |
| Koronis | 0.2575 | 0.0027* | - |
| Minnewaska | 0.3106 | 0.0108* | 0.1337 |

*indicates significance at the 99% confidence level.

**indicated significance at the 95% confidence level

^1^Indicator variables are *Fishing* (1: Fishing chosen as primary reason for visit; 0: All others*), Gender* (1:Female; 0:Male), *Local* (1: respondents indicated they were staying at/coming from home; 0: respondents indicated they were not), *Gender* (1: female; 0: male); no respondents choose non-binary.  *Aged 45 or greater* (1: respondent is 45 years of age or older; 0: respondent is less than 45), and *AIS Knowledge* (1: Correctly identified AIS in the lake; 0: Did not). Scalar variables are *Income* (1 through 12; 1: Income < $20,000, 2: $20,000 ≤ Income ≤ $29,999, 3: $30,000 ≤ Income ≤ $39,999, 4: $40,000 ≤ Income ≤ $49,999,5: $50,000 ≤ Income ≤ $59,999, 6: $60,000 ≤ Income ≤ $69,999, 7: $70,000 ≤ Income ≤ $79,999, 8: $80,000 ≤ Income ≤ $89,999, 9: $90,000 ≤ Income ≤ $99,999, 10: $100,000 ≤ Income ≤ $149,999, 11: $150,000 ≤ Income ≤ $199,999, 12: Income ≥ $200,000), *Awareness of AIS Problem* (0: Not a problem at all, 1: Slight Problem, 2: Moderate Problem, 3: Severe Problem), *Perceived AIS* *Risk* is the sum across the risk categories, where the options were (1:No risk at all, 2: Slight risk, 3: Moderate risk, 4: Extreme Risk). ), *Visit Motivation* measured how important it was for the respondent to “be on my own” (0: Not at all important, 1: Slightly important, 2: Moderately important, 3: Very important, 4: Extremely important), and *Education* (1: Did not complete high school, 2: Completed high school, 3: Some college but no degree, 4:Associate degree or vocational degree, 5: College bachelor’s degree, 6:Some postgraduate work but no degree, 7: Completed graduate degree).
