# Supplementary material for: Recreationist willingness to pay for aquatic invasive species management
Source: PLoS One. 2021 Apr 14;16(4):e0246860. doi: 10.1371/journal.pone.0246860 (PMC8046257; doi:10.1371/journal.pone.0246860)
Supplement: S3 Appendix table — (DOCX) [file pone.0246860.s006.docx]

**S3 Appendix table.** Variable correlation matrix (Spearman’s).

|  | Income | Gender | Edu-cation | Age (45+) | Local | On Own | Fish-ing | AIS Prob | AIS Know | AIS Risk | |
| --- | --- | --- | --- | --- | --- | --- | --- | --- | --- | --- | --- |
| Income | 1 |  |  |  |  |  |  |  |  |  | |
| Gender | -0.119 | 1 |  |  |  |  |  |  |  |  | |
| Education | 0.412 | 0.093 | 1 |  |  |  |  |  |  |  | |
| Age (45+) | 0.170 | -0.089 | 0.101 | 1 |  |  |  |  |  |  | |
| Local | -0.255 | -0.038 | -0.170 | -0.140 | 1 |  |  |  |  |  | |
| On Own | -0.043 | -0.055 | -0.034 | 0.117 | -0.035 | 1 |  |  |  |  | |
| Fishing | -0.101 | -0.265 | -0.194 | -0.021 | 0.140 | 0.109 | 1 |  |  |  | |
| AIS Problem | -0.019 | 0.077 | 0.036 | 0.064 | -0.102 | 0.033 | -0.071 | 1 |  |  | |
| AIS Knowledge | 0.053 | -0.121 | 0.004 | 0.023 | 0.080 | 0.023 | 0.128 | -0.041 | 1 |  | |
| AIS Risk | 0.007 | 0.210 | 0.067 | 0.069 | -0.122 | 0.083 | -0.123 | 0.540 | -0.071 | 1 | |
|  |  |  |  |  |  |  |  |  |  |  | |
|  |  |  |  |  |  |  |  |  |  |  | |
|  | 0.1 <= Coef < 0.2 | | | 0.2 <= Coef < 0.3 | | | 0.3 <= Coef | | |  |  |
