# Supplementary material for: Recreationist willingness to pay for aquatic invasive species management
Source: PLoS One. 2021 Apr 14;16(4):e0246860. doi: 10.1371/journal.pone.0246860 (PMC8046257; doi:10.1371/journal.pone.0246860)
Supplement: S4 Appendix table — (DOCX) [file pone.0246860.s007.docx]

**S4 Appendix table.** Estimated willingness to pay for responders in preferred model and non-responders

|  |  | Non-responders | |  |  | Responders (Preferred Model) | |
| --- | --- | --- | --- | --- | --- | --- | --- |
|  | n | WTP | CI (95%) |  | n | WTP | CI (95%) |
| All | 167 | $7.19 | $5.48 to $8.89 |  | 538 | $9.87 | $9.07 to $10.67 |
| Koronis | 22 | $5.62 | $2.27 to 8.96 |  | 102 | $10.40 | $9.28 to 11.51 |
| Minnewaska | 59 | $9.64 | $4.47 to 10.64 |  | 185 | $10.34 | $9.27 to 11.40 |
| Pokegama | 39 | $5.69 | $1.45 to 9.93 |  | 103 | $8.96 | $7.77 to 10.14 |
| Gull | 47 | $8.61 | $5.65 to 11.55 |  | 148 | $9.56 | $8.38 to 10.74 |
